# Antimicrobial susceptibility of *Rhodococcus equi* strains isolated from foals in Chile

**DOI:** 10.1007/s11274-023-03677-2

**Published:** 2023-06-22

**Authors:** María Paz Zúñiga, Elena Badillo, Pedro Abalos, Eduardo David Valencia, Pedro Marín, Elisa Escudero, Juan Sebastian Galecio

**Affiliations:** 1 Facultad de Agronomía y Veterinaria, Universidad Nacional de Rio Cuarto, Córdova, Argentina; 2 Escuela de Medicina Veterinaria, Facultad de Ciencias, Universidad Mayor, Santiago de Chile, Chile; 3 Department of Pharmacology, Faculty of Veterinary Medicine, University of Murcia, Murcia, 30100 Spain; 4 Facultad de Ciencias Veterinarias y Pecuarias, Universidad de Chile, La Pintana, 8820808 Chile; 5 Instituto de Microbiología, Colegio de Ciencias Biológicas y Ambientales, Universidad San Francisco de Quito, Quito, EC 170157 Ecuador; 6 Colegio de Ciencias de la Salud, Escuela de Medicina Veterinaria, Universidad San Francisco de Quito, Quito, EC 170157 Ecuador

**Keywords:** Antimicrobial susceptibility, Foals, Macrolides, *Rhodoccocus equi*

## Abstract

*Rhodococcus equi* is responsible for foal pneumonia worldwide, with a significant economic impact on the production and breeding of horses. In Chile, the first case was reported in 2000, and since then, its incidence has been increasing. Distinctive characteristics of *R. equi* as an intracellular pathogen in macrophages, emergence of virulence plasmids encoding surface lipoprotein antigens, and appearance of antibiotic resistance against macrolides and rifampicin have significantly complicated the treatment of *R. equi* pneumonia in foals. Therefore, in vitro susceptibility studies of first-line and newer antibiotics against *R. equi* are the first step to establishing effective treatments and optimizing new therapeutic options. The aim of the present study is to determine the susceptibility profile of fourteen strains of *R. equi* isolated from foals in Chile to several antibiotics of the macrolide group including azithromycin, amikacin, tildipirosin and gamithromycin as well as others such as rifampicin, doxycycline and ceftiofur. Identification of *R. equi* in collected isolates from foals in Chile has been performed by CAMP test and PCR based on detecting of the gene encoding the 16 S rRNA. The presence of genes encoding virulence plasmids was also determined using PCR. Results obtained have demonstrated presence of virulent *R. equi* strains in Chile. In vitro susceptibility pattern to different antibiotics has shown better results for doxycycline and rifampicin similar to previous studies performed. Current macrolides have been evaluated in order to consider alternative treatment options in a context of emerging resistance to classic macrolides and rifampicin, obtaining better results with gamithromycin (MIC range of 0.125 to 128 mg/ml) than with tildipirosin (MIC range of 16 to 128 mg/ml). An adequate diagnosis of bacterial susceptibility based on antibiograms is necessary to treat the *Rhodococcus equi* infection in foals.

## Introduction

*Rhodococcus equi* (*R. equi*) pneumonia is a primary respiratory disease in foals worldwide and is considered a significant pathogen in the equine breeding period (Takai et al. 1995). *Rhodococcus equi* may be considered a globally distributed microorganism in many countries, such as Chile, where it was first reported in 2000 (Paredes et al. 2000). The finding of *R. equi* in the country suggests that the infection is widespread, causing severe animal health complications considering that it causes a high morbidity rate and a poorer prognosis in foals where macrolide-resistant isolates are found (Cohen 2014). It is a ubiquitous pathogen with a high capacity to survive under extreme temperatures environment and dirty areas, conditions associated with the natural breeding seasons in horses during spring and summer (Giguère et al. 2012; Gressler et al. 2018). The different routes of transmission, difficulty of early diagnosis, and progressive increase in *R. equi* incidence make it one of the most worrying pathogens for foals worldwide particulary in intensive breeding programs (Paredes et al. 2000; Takai et al. 1995).

Foaling season is the most sensitive period in the horse industry due to the occurrence of the highest rate of illness at this time, which can lead to repercussions on the health and well-being of adult horses (Franco-Ayala and Oliver-Espinosa 2015). In this sense, *R. equi* has a significant impact on this industry by causing economic losses not only in terms of deaths but also in growth retardation, poor performance in sport horses, expensive veterinary treatment protocols, and decreasing value of horses from endemic areas (Martens et al. 1990; Peiró et al. 2002). Foals are particularly susceptible to *R. equi*, mainly under six months of age but less often may also be immunodeficient adult horses (Paredes et al. 2000). Moreover, *R. equi* infection is also described as a zoonosis in humans and has gained importance in recent years as a cause of pneumonia, mainly in immunocompromised patients (Byrne et al. 2001; Vázquez-Boland et al. 2013). *Rhodococcus equi* colonizes the respiratory system, producing pyogranulomatous pneumonia that often becomes chronic, producing abscesses in the lung, mesenteric and bronchial lymph nodes (Prescott 1991). Respiratory infections are often complicated in about 50% of cases with intestinal infection (Yager 1987), especially in foals under 1 to 3 months of age, whose immune system has not yet fully matured (Paredes et al. 2000). Recent studies also demonstrate that ill foals extrapulmonary infections may occur, including abdominal abscess, colitis, osteomyelitis, and immune-mediated disorders such as non-septic synovitis and uveitis (Le Corre et al. 2021; Rakowska et al. 2022; Reuss et al. 2009).

Virulence of *R. equi* is based on the ability to replicate inside macrophages, which de-pends on its capacity to interfere with endosomal maturation after phagocytosis (Hondalus and Mosser 1994). Survival inside cells is related to bacterial capacity to avoid destruction by the phagolysosome. This quality is connected to plasmids gene expressions of virulence-associated proteins (VAPs), present in certain strains isolated from foals with *R. equi* infection, which allows the bacterial growth necessary to ensure the development and progression of the disease (Takai 1997). Although there are several VAPs, virulence-associated protein A (vapA) is the most studied due to its more robust association with virulent equine strains. The virulence plasmids encoding for vapA are necessary for the pathogenicity associated with the replication of the microorganism inside macrophages and their enhanced cytotoxicity (Giguère 2010).

*Rhodococcus equi* is often sensitive to many antimicrobials in vitro, but its antimicrobial susceptibility in vivo does not correlate adequately in all cases, making the effect of some antimicrobials with insufficient penetration into tissues and lung defense cells ineffective (Muscatello 2012; Sweeney et al. 1987). Macrolide antibiotics have shown extensive disposition in respiratory tissue and epithelial lining fluid (Jacks et al. 2001) and desirable inhibitory capacity against *R. equi* compared to other groups of antimicrobials. Combining a macrolide (clarithromycin, erythromycin, or azithromycin) with rifampicin is the recommended treatment for *R. equi* infection, based on data of its in vitro activity and retrospective clinical trials on its use (Cisek et al. 2014; Reuss et al. 2009). However, pharmacokinetic studies show drug interactions with a decreased disposition of macrolides in lung tissues (Berlin et al. 2016).

In several cases, erythromycin can increase minimal inhibitory concentration (MIC) values (Fenton and Buckley 2015) and is associated with many adverse effects. Advantages of azithromycin and clarithromycin use over erythromycin include better oral bioavailability, prolonged half-life, and higher concentrations in bronchoalveolar cells and lung epithelium-associated fluid in foals (Davies et al. 2002; Jacks et al. 2003; Suarez-Mier et al. 2007). One adverse effect described by macrolide therapy is the development of diarrhea (Giguère 2010). Although a small percentage of them require fluid therapy, this adverse reaction is a problem due to the limited range of efficient alternative drugs. In this context, oral administration of doxycycline in combination with rifampicin or macrolides has an in vitro synergism and could be successful in the treatment of *R. equi* pneumonia. An alternative for treating rifampin-resistance isolate could be to combine doxycycline with a macrolide (Giguère 2010; Sweeney et al. 1987).

A difficulty in treating infection caused by *R. equi* is the emergence of macrolide-resistance strains, which have been studied for 20 years (Burton et al. 2015; Fenton and Buckley 2015; Giguère 2010), considering limited compound options and the high mortality associated with macrolide-resistance *R. equi* (Giguère et al. 2010). Using newer macrolides such as tildipirosin or gamithromycin alone or combined with rifampicin may be an option for treatment. In any case, rational use of antimicrobial therapy is necessary, including identifying the pathogen by cultures, PCR, and bacterial susceptibility testing to determine the most effective treatment with the lowest risk. This study was designed to obtain in vitro susceptibility values of pathogenic *R. equi* strains to azithromycin, rifampicin, ceftiofur, doxycycline, amikacin, tildipirosin, and gamithromycin based on their MIC values in order to contribute to the development of an effective treatment for *R. equi* infection in foals in Chile.

## Materials and methods

### Animals and sample collection

The samples used in this study were submitted through equine veterinary practitioners in Chile related to Thoroughbred breeding farms with suspected *R. equi* infection in foals from November 2019 to January 2021. Samples were collected from *post-mortem* lung lesions or obtained from deep nasopharyngeal swabs or transtraqueal aspirates. All collected samples were transported in Stuart medium, stored in isothermal boxes at 4 °C, and immediately analyzed. Inclusion criteria to consider a suspicious case of *R. equi* infection was defined as a foal showing one or more of the following clinical findings: lower respiratory tract disease signs, cytological evidence of septic airway inflammation, and radiographic/ultrasonographic evidence of pneumonia (Giguère et al. 2011). In addition, diarrhea was also considered as an inclusion criterion since there is evidence that it is an important sign of extrapulmonary disorders caused by *R. equi* infection in foals (Giguère et al. 2011, Giguère 2012).

### Isolation and characterization of ***Rhodocuccus equi***

The samples were cultured on trypticase soy agar plates with 5% sheep blood and incubated at 37ºC. The development of suspicious colonies was observed until 72 h of incubation, which were subsequently subjected to a Chris-tie-Atkins-Munch-Peterson test (CAMP test), where a streak of the suspected *R. equi* strain was faced perpendicularly with one of *Staphylococcus aureus*. This test verified the synergy in the hemolytic activity of *Staphylococcus aureus* beta hemolysin and *Rhodococcus equi* factor (Camponovo and García 2006). It was established that fourteen strains were positive for the CAMP test. Subsequently, these strains were subjected to replicate PCR detecting of ARN16s (458 bp product) and identification of the main factor associated with virulence vapA (564 bp product) (Krewer et al. 2008). Of the fourteen isolated and identified strains, the presence of vapA was confirmed in twelve. The two vapA-negative strains were categorized as non-pathogenic *Rhodococcus equi*, but since they originated from animals with pulmonary lesions consistent with *Rhodococcus equi* bronchopneumonia, it was decided to include these isolates in the antimicrobial susceptibility evaluation.

### Susceptibility testing

Azithromycin, tildipirosin, gamithromycin, rifampicin, doxycycline, amikacin, and ceftiofur were selected for susceptibility testing. Antibiotics were dissolved in appropriate solvents and diluted in sterile distilled water to prepare stock solutions following the guidelines by Clinical and Laboratory Standards Institute (2008). Minimum inhibitory concentrations of the fourteen isolates of *R. equi* were obtained by the microdilution broth technique. Serial two-fold dilutions of the antimicrobial agents were prepared from the stock solution with concentrations ranged from 0.03 to 128 mg/L for all antibiotics. Serial dilutions were prepared using cation-adjusted Mueller–Hinton broth with 2.5% defibrinated horse blood. Inocula were produced by diluting the overnight culture of *R. equi* in cation-adjusted Mueller–Hinton broth with a buffered saline solution to a density of 0.5 on McFarland Turbidity Scale. This solution was diluted 20-fold and inoculated into each microtiter well. Finally, plates were incubated at 36 °C and observed at 24 h. Plates were run in an ELISA microplate reader (ELx808TM, Biotek® Instruments, Inc.), absorbance was measured at 490 nm wave-length. Each read was setup using the Gen 5 Reader Control (Biotek® Instruments, Inc.), the data output was exported automatically to Excel for further statistics analysis. The reference strain *Escherichia coli* (ATCC 25,922) was used as control.

### Statistical analysis

Frequency of *Rhodococcus equi* strains in relation to their minimal inhibitory concentrations and median, MIC_50_, and MIC range values of azithromycin, rifampicin, ceftiofur, doxycycline, amikacin, tildipirosin, and gamithromycin were tabulated.

## Results

### ***Rhodococcus equi*** characterization

A total of fourteen isolates from foals in Chile were collected and confirmed as *R. equi* by PCR by identification based on sequencing of the gene encoding the 16 S rRNA. Confirmation of the identification of the bacteria was also done by CAMP test. All bacteria from foal isolates were confirmed as *Rohdococcus equi*. Of the fourteen isolated and identified strains, the presence of vapA was confirmed in twelve.

### Minimal inhibitory concentrations of antibiotics againts ***R. equi*** isolates

The specific MIC values for each selected antibiotic agent may be observed in Table 1 where is presented the median, MIC values of these antibiotics that inhibited 50% of the isolates of *Rhodococcus equi* (MIC_50_), and minimum and maximum MIC values (range).

**Table 1:** Antibacterial susceptibility of *Rhodococcus equi* (n = 14) to different antibiotics for the treatment of *Rhodococcus equi* infection in foals

Antibiotics	Number of strains with MIC (µg/L)													Median (µg/mL)	MIC_50_ (µg/mL)	MIC range (µg/mL)
	0.03	0.06	0.12	0.25	0.5	1	2	4	8	16	32	64	128			
**Amikacin**	-	-	-	-	-	-	-	4	8	2	-	-	-	8	8	4–16
**Azithromycin**	-	-	-	-	10	1	-	-	1	-	-	-	2	0.5	0.5	0.5–128
**Ceftiofur**	-	-	-	-	-	-	1	4	2	5	-	1	1	12	8	2-128
**Doxycycline**	-	-	-	3	9	1	-	1	-	-	-	-	-	0.5	0.5	0.25-4
**Gamithromycin**	-	-	1	-	2	8	1	-	-	-	-	-	2	1	1	0.12–128
**Rifampicin**	2	5	2	-	2	-	-	1	-	-	-	-	2	0.0925	0.06	0.03–128
**Tildipirosin**	-	-	-	-	-	-	-	-	-	6	5	-	3	32	32	16–128

Our best results were obtained from rifampicin and doxycycline. Doxycycline shows a MIC range of 0.25 to 4 mg/ml and a MIC_50_ of 0.5 mg/mL. In this case, from fourteen strains tested, only one exhibited MIC values above 1 mg/mL. Rifampicin shows the best results for MIC_50_ with a value of less than 0.03 mg/mL. Although many of the strains studied show MIC values below 1 mg/mL, two of the strains studied are above 128 mg/mL. Among the macrolides studied, azithromycin (MIC_50_ = 0.5 mg/mL) and gamithromycin (MIC_50_ = 1 mg/mL) show similar results, except in the case of tildipirosin, which presents the worst results with high MIC values (MIC_50_ = 32 µg/mL) for all the strains studied.

## Discussion

The identification of *R. equi* has been positive in fourteen isolates obtained and con-firmed by 16 S rRNA analysis. This method is more sensitive than standard identification testing, as indicated by some publications (Kirkan et al. 2021; Vivrette et al. 2000). Confirming the presence of VAPs genes in *R. equi* allows an understanding of the virulence mechanisms of this pathogen (Takai 1997; Willingham-Lane et al. 2016). Thus, the presence of VAPs genes, mainly vapA has been associated with an increased mortality rate of *R. equi* in foals, providing a reference for epidemiological studies (Kirkan et al. 2021). In our study, twelve of the fourteen (12/14) *R. equi* isolates had VAPs genes encoding specific antigens virulence plasmid. Only two of the isolates were classified as non-virulent strains. In this way, the presence in Chile of virulent variants of *R. equi* in foals is confirmed, as previous studies showed (Paredes et al. 2000; Lohse et al. 2019).

The rational use of antimicrobials for treating *R. equi* in foals involves a thorough in vitro antibiotic susceptibility study. Considering that the main therapeutic options are based on using macrolide alone or in combination with rifampicin and doxycycline (Giguère 2017; Prescott 1991; Muscatello 2012), evaluating in vitro effect of first-line and newer antimicrobials is crucial to determine the potential effectiveness of the treatments against *R. equi* infection. This is especially important, considering the development of *R. equi* isolates resistance against macrolides and rifampicin, which has been extensively reported in recent years (Cisek et al. 2014; Kirkan et al. 2021; Willingham-Lane et al. 2016).

Azithromycin reaches and maintains a high concentration in foal alveolar macrophages, showing desirable outcomes after administration in foals (Davies et al. 2002). Values reported in our study indicate a MIC_50_ of 0.5 mg/ml, similar to those obtained in previous studies (Jacks et al. 2003; Giguère 2017, Gressler et al. 2015). Our results have been better than other researches (Kirkan et al. 2021; Riesenberg et al. 2014), only two strains showed high MIC values (128 mg/mL) for azithromycin and most of the investigated macrolides.

Tildipirosin and gamithromicin are parenteral macrolides indicated for treatment of respiratory diseases. Recent studies demonstrate its high distribution, bioavailability, and safety in horses (Galecio et al. 2022) and foals (Berghaus et al. 2012; Berlin et al. 2017). Results obtained for tildipirosin have been unfavorable than other macrolides investigated, with a MIC range of 16 to 128 mg/ml. There are not found previous in vitro susceptibility studies for gamithromycin. However, its efficacy in treating *R. equi* pneumonia in foals alone or in combination with rifampicin has been demonstrated in different reports with similar results to other macrolides (Hildebrand et al. 2015).

Combination of erythromycin-rifampicin was a choice for treating *Rhodococcus equi* infection in foals for a long time since the 1980s (Sweeney et al. 1987) but has been replaced by the com-bination of other macrolides with rifampicin because of its efficacy and increased safety (Burton et al. 2013a, b; Giguére et al. 2004). However, combination of macrolides with rifampicin has been shown to have a drug interaction in vivo with a lower tissues disposition of macrolides, which can promote the development of antibiotic resistance (Berlin et al. 2016). Several studies have shown resistance to macrolides and rifampicin in foals with *R. equi* associated with the wide-spread use of these antibiotics (Burton et al. 2013a, b; Cisek et al. 2014; Giguère and Cohen 2018; Giguère et al. 2010; Giguère et al. 2017; Riesenberg et al. 2014). The therapy of foals infected with *R. equi* resistant to macrolides and rifampicin should be further investigated, due to the limited therapeutic options currently available.

Rifampicin should always be used with another antimicrobial to avoid the risk of emergence resistance (Cicek et al. 2014; Fenton and Buckley 2015). The MIC_50_ results for rifampicin were similar to those reported in other studies (Kalinowski et al. 2020, Nordmann and Ronco 1992) and as with azithromycin, two isolates showed MIC levels of 128 mg/mL. In vitro susceptibility values for doxycycline in our study have shown the best results of all the tested antibiotics with MIC range of 0,25 − 4 mg/mL and all susceptible isolates. The obtained data are moderately superior compared to data reported by previous studies (Jacks et al. 2003; Riesenberg et al. 2014; Berghaus et al. 2015; Kalinowski et al. 2020, Nordmann and Ronco 1992). In this context, doxycycline, in combination with rifampicin, may be an option due to the synergy they could present when concomitant administration is used (Giguère et al. 2012).

Ceftiofur, a third-generation cephalosporin, has shown efficacy against *R. equi in vitro* (Jacks et al. 2003). With regard to its pharmacodynamics, previous in vitro susceptibility studies demonstrate better values with MIC range values between 0.5 and 2 mg/ml (Jacks et al. 2003), 1 to 4 mg/ml (Erol et al. 2020), and 0.25 to 16 mg/mL (Riesenberg et al. 2014). Susceptibility results for amikacin show an MIC range of 4 to 16 mg/ml. Similar results were found in previous studies (Erol et al. 2020) with a range of 4 to 32 mg/mL. Other susceptibility studies for amikacin showed improved results against *R. equi* (Jacks et al. 2003; Kalinowski et al. 2020, Nordmann and Ronco 1992).

## Conclusions

The present study corroborates the presence of virulent strains of *R. equi* in foals in Chile, whose sensitivity to different antibiotics is adequate, presenting better antibiotic susceptibility for doxycycline, azithromycin, and rifampicin. Of the fourteen isolates studied, two showed high MIC values suggesting resistance to different macrolides and also to rifampicin.

New macrolides such as tildipirosin and gamithromicyn should be tested more extensively and with a higher number of isolates to determine the in vitro susceptibility both alone and in combination with other antibiotics to establish its clinical efficacy.

The present context of emerging resistance to macrolides and rifampicin makes an adequate diagnosis of bacterial susceptibility necessary based on antibiogram studies before establishing an adequate treatment of *Rhodococcus equi* infection.

## Data Availability

The data that support the findings of this study are openly available in Science Data Bank at 10.57760/sciencedb.07119 published on 2023–01–11.
